# Non-fasting lipids detection and their significance in pregnant women

**DOI:** 10.1186/s12944-019-1038-z

**Published:** 2019-04-11

**Authors:** Yulong Li, Jianxun He, Xiaoli Zeng, Song Zhao, Xuebing Wang, Hui Yuan

**Affiliations:** Department of Clinical Laboratory, Beijing Anzhen Hospital, Capital Medical University, Anzhen Road No. 2, Chaoyang District, Beijing, People’s Republic of China

**Keywords:** Pregnancy, Lipid, Lipoprotein, Non-fasting, Gestational hyperlipidaemia

## Abstract

**Background:**

The majority of pregnant women present an increase in lipids. To investigate the influence of the non-fasting state in the lipid and lipoprotein profile in pregnancy, we have aimed to assess the dynamic change of serum lipid and lipoprotein profile with serum glucose in pregnancy to contrast the differences between fasting and non-fasting state.

**Methods:**

Forty-five pregnant women and 41 controls were included in our study. All serum samples were assayed for TC, TG, HDL-C, LDL-C, ApoB, ApoA-1, Lp(a), sdLDL, and Glu concentrations. The comparison between pregnant women and controls (fasting and 2 h after breakfast), differences of these measurement results at three point-in-time, the associations between the concentrations of serum lipid and some maternal and fetus characteristics was conducted with statistical analysis.

**Results:**

Except Glu (*p* < 0.001), there were no significant differences of all lipids between three point-in-time in pregnant women (*p* > 0.1). The statistically higher levels were found in fasting TC (*p* = 0.003), TG (*p* = 0.019), LDL-C (*p* = 0.002), ApoB (*p* = 0.001), ApoA1 (*p* = 0.013) and sdLDL (*p* < 0.001) of pregnant women compared with controls. Besides, the statistically significances were also found in 2-h TC (*p* = 0.001), LDL-C (*p* = 0.001), ApoB (*p* < 0.001), Glu (*p* = 0.013), ApoA-1 (*p* = 0.009) and sdLDL (*p* < 0.001) of pregnant women compared with controls. Otherwise, in non-fasting status (2 h after breakfast), pregnancy complication was relevant to TC (*p* = 0.041), HDL-C (*p* = 0.014), Glu (*p* = 0.004). Delivery mode was relevant to TC (*p* = 0.012), HDL-C (*p* = 0.013), LDL-C (*p* = 0.026), ApoA-1 (*p* = 0.012), and sdLDL (*p* = 0.044). BMI was relevant to TG (*p* = 0.027).

**Conclusion:**

We have suggested the non-fasting lipids detection can be used for estimate lipid metabolism in pregnant women.

## Introduction

Pregnancy is characteristic of physiological metabolic adaptations [1, 2]. Pregnant women experience peripheral insulin resistance, and levels of hormones increase compared with the non-pregnant state [3, 4]. Consequently, all serum lipid fractions progressively increase [5, 6]. These metabolic adaptations are essential to support adequate fetal growth and development [1, 2]. The change of serum lipid fractions is physiological and results from increased insulin resistance, lipoprotein synthesis and lipolysis in adipose tissue which mobilize fats to serve as an energetic supply for fetal growth [7, 8]. The majority of pregnant women presents an increase in triglycerides (TG), high density lipoproteins (HDL), and low density lipoproteins (LDL) [9]. Moreover, in some pregnant women, total cholesterol (TC) may increase by 25 to 50% and TG by 200 to 400% [10, 11].

Changes in lipid metabolism have been shown to ensure a continuous supply of nutrients to the fetus, despite the intermittent maternal food intake [12]. However, the disturbances of lipid during pregnancy may also lead to the high risk of cardiovascular disease and result in undesirable outcomes for both the mother and the fetus [6, 13], such as preeclampsia [13, 14], gestational diabetes mellitus [15, 16], intrauterine growth restriction and premature birth [6, 17]. Some previous studies have showed that the most dramatic damage in the lipid and lipoprotein profile in normal pregnancy is serum TG, which may be as high as two or three folds [18].

Recently, more and more researchers have focus on the relationship between abnormal serum lipid and lipoprotein profile and metabolic diseases in pregnancy. In these researches, non-fasting blood sampling was performed to study the lipid and lipoprotein profile in pregnancy [19–21]. They believed the non-fasting state may have diluted their results slightly but no data was offered to support it. As most of the day is spent in a non-fasting state, non-fasting lipid levels may predict health risks even more accurately [22]. Hence, the influence of the non-fasting state in the lipid and lipoprotein profile in pregnancy is essential to exclude the interference of intake. Unfortunately, no data has been shown in present.

Recently, researches indicated that the high risk of cardiovascular disease was related to non-fasting lipid, including triglycerides, apolipoprotein B and non-HDLC, instead of fasting lipid [23, 24]. Non-fasting triglycerides were reported to have a greater impact on risk of cardiovascular diseases than fasting triglycerides [25, 26]. Since 2009, non-fasting lipid detection has become the clinical standard in Denmark, based on recommendations from the Danish Society for Clinical Biochemistry [27, 28]. Furthermore, American College of Cardiology (ACC)/American Heart Association Guideline (AHA) re-examined the need for fasting lipid measurements in various clinical scenarios, and considerednon-fasting lipid detection could be used to evaluate the risk of arteriosclerotic cardiovascular disease in the 2013 [29]. Since 2014, the UK National Institute of Clinical Excellence (NICE) guidelineshave endorsed non-fasting lipid testing in the primary prevention of cardiovascular disease [30]. Then, in 2016, the European Atherosclerosis Society/EuropeanFederation of Clinical Chemistry and Laboratory Medicine (EAS/EFLM) joint consensus statement proposes recommendations on that fasting is not required routinely for assessing the lipid profile while consideration should be given to repeating the fasting lipid testing when non-fasting triglyceride is more than 5 mmol/L [31].

In this study, we have aimed to assess the dynamic change of serum lipid and lipoprotein profile with serum glucose in pregnancy to contrast the differences between fasting and non-fasting state. At the same time, we have also attempted to advance understanding and provide more potent evidence about the relationship between the non-fasting lipid and lipoprotein profile and the maternal and infant pathological status.

## Materials and methods

### Study population

Pregnant women who attended regular prenatal health care in Beijing Anzhen Hospital were invited to participate in this study. The inclusion criteria of pregnant women included: a) pregnant at 20–30 gestational weeks; b) had integrated medical records and clear gestational age; c) singleton pregnancy; and d) naturally fertilized. The exclusion criteria of pregnant women included: a) multiple pregnancy; b) had diabetes mellitus, hypertension, cardiovascular diseases, chromosomal abnormalities, inherited metabolic diseases or thyroid diseases before pregnancy; c) experienced serious infection during early pregnancy; d) experienced drug therapy; and e) fertilized with assisted reproductive techniques. A total of 45 expectant pregnant women were recruited (Table 1). All data about maternal age, height, gestational weight, lifestyle, education background, residence and other important information was collected at the first time of their coming. They were followed from recruitment to delivery. Information on delivery mode, gestational age, birth weight, Apgar scores and perinatal outcomes were recorded by obstetricians upon delivery. As a control group, 41 age-matched, healthy non-pregnant women were included in this study. Our study was approved by the hospital’s Clinical Research Ethics Committee in Beijing Anzhen hospital and written informed consent was obtained from all patients prior to serum sampling.

**Table 1:** Clinical information of pregnant women

	Pregnant women
Average age (year, mean ± standard deviation)	30.7 ± 3.8
BMI (mean ± standard deviation)	26.7 ± 3.4
Detective trimester	second
Delivery mode	
Cesarean	26
Forceps Delivery	1
Eutocia	18
Pregnancy complication	13
Labour time (week, mean ± standard deviation)	38.2 ± 1.9
Perinatal outcomes	
Healthy	31
Premature	13
Stillbirth	1
Apgar scores (median, 95% confidence interval)	10 (2, 10)

### Serum lipids measurement

Venous blood samples were taken after overnight fasting from all the participants. Venous blood samples were taken again at 1 and 2 h after having breakfast. All the volunteers have taken the same breakfast with about 400 Calorie. The blood samples were collected in a 5 mL vacutainer (BD, US) for the preparation of serum. 1.5 mL aliquots of serum samples were obtained by centrifugation (3500 rpm for 10 min) and stored at − 20 °C until analysis. Every sample was assayed for TC, TG, HDL-C, LDL-C, apolipoprotein B (ApoB), apolipoprotein A-1(ApoA-1), Lipoprotein (a) (Lp(a)), small dense LDL (sdLDL), and glucose (Glu) concentrations. All the measurements were performed on an automatic biochemical analyser (AU5400, Beckman, US) respectively with TC, TG, HDL-C, LDL-C, ApoB, ApoA-1, Lp(a), sdLDL, and Glu detection kits (Beckman, US). All measurements were assayed with continuous monitoring method and quality controls were appropriate before these assays.

### Statistical analysis

Data of the concentrations of TC, TG, HDL-C, LDL-C, ApoB, ApoA-1, Lp(a), sdLDL, and Glu was expressed as mean ± standard deviation (SD). The comparison between pregnant women and controls (fasting and 2 h after breakfast) was conducted with independent-samples t-test. Differences of these measurement results at three point-in-time were also compared statistically with One-Way Anova. Spearman’s nonparametric correlation analysis were applied to explore the associations between the concentrations of serum lipid (fasting and 2 h after breakfast) and some characteristics such as pregnancy complications, perinatal outcomes, delivery mode, gestational age, and Apgar scores. The correlation between levels of serum lipid (fasting and 2 h after breakfast) and other characteristics involving maternal age and Body Mass Index (BMI) were investigated by Pearson correlation analysis. Probability values of less than 0.05 were considered statistically significant. All statistical analyses were carried out with SPSS software version 17.0.

## Results

**Fig. 1:**
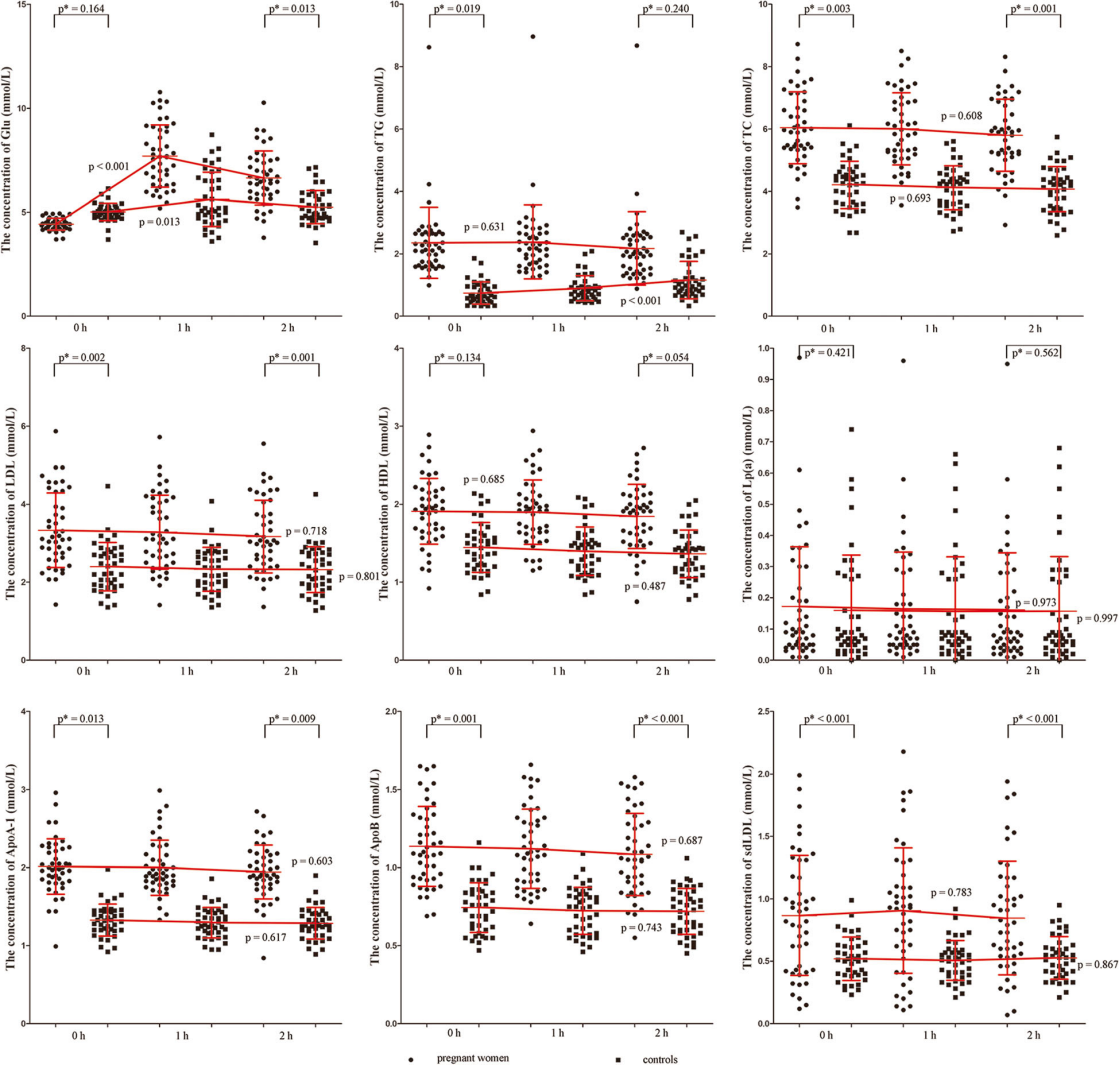
The concentrations of TC, TG, HDL-C,LDL-C, ApoB, ApoA-a, Lp(a), sdLDL, and Glu of pregnant women and controls at different time-points. The differeneces were shown as *p*-value

Results of the concentrations of TC, TG, HDL-C,LDL-C, ApoB, ApoA-a, Lp(a), sdLDL, and Glu of pregnant women and controls at different points are illustrated graphically in Fig. 1. The median levels of these lipids are shown in Table 2. Except Glu (*p* < 0.001), there were no significant differences of all lipids between three point-in-time in pregnant women (*p* > 0.1) (Fig. 1). Glu and TG were found a significant change (*p* = 0.013 and *p* < 0.001) between three point-in-time in controls, and other lipid parameters were found without a significant differences in controls (*p* > 0.1) (Fig. 1). The statistically higher levels were found in fasting TC (*p* = 0.003), TG (*p* = 0.019), LDL-C (*p* = 0.002), ApoB (*p* = 0.001), ApoA1 (*p* = 0.013) and sdLDL (*p* < 0.001) of pregnant women compared with controls (Fig. 1). Besides, the statistically significances were also found in 2-h TC (*p* = 0.001), LDL-C (*p* = 0.001), ApoB (*p* < 0.001), Glu (*p* = 0.013), ApoA-1 (*p* = 0.009) and sdLDL (*p* < 0.001) of pregnant women compared with controls (Fig. 1).

**Table 2:** The median levels of lipids of pregnant women and controls

	Pregnant women			Controls		
	Fasting	1 h	2 h	Fasting	1 h	2 h
CHOL	6.04 ± 1.14	6.01 ± 1.15	5.80 ± 1.14	4.21 ± 0.76	4.12 ± 0.70	4.08 ± 0.72
TG	2.35 ± 1.13	2.39 ± 1.17	2.18 ± 1.17	0.74 ± 0.35	0.89 ± 0.40	1.16 ± 0.60
HDLD	1.91 ± 0.42	1.90 ± 0.41	1.84 ± 0.41	1.45 ± 0.32	1.39 ± 0.31	1.36 ± 0.31
LDLD	3.33 ± 0.95	3.28 ± 0.94	3.17 ± 0.92	2.40 ± 0.62	2.33 ± 0.57	2.32 ± 0.59
APOB	1.14 ± 0.25	1.12 ± 0.25	1.08 ± 0.26	0.74 ± 0.16	0.72 ± 0.15	0.72 ± 0.15
Glu	4.43 ± 0.28	7.70 ± 1.48	6.65 ± 1.28	5.02 ± 0.42	5.62 ± 1.30	5.25 ± 0.79
Lpa	0.17 ± 0.19	0.16 ± 0.18	0.16 ± 0.18	0.16 ± 0.18	0.16 ± 0.17	0.16 ± 0.17
APOA-1	2.01 ± 0.35	2.00 ± 0.35	1.94 ± 0.34	1.33 ± 0.21	1.29 ± 0.20	1.29 ± 0.20
sdLDL	0.87 ± 0.48	0.90 ± 0.50	0.85 ± 0.45	0.52 ± 0.17	0.51 ± 0.16	0.53 ± 0.17

The correlation between serum lipid and characteristics of pregnant women and neonates was shown in Table 3. In fasting status, pregnancy complication was relevant to TG (*p* = 0.042) and Glu (*p* = 0.014). Delivery mode was relevant to TC (*p* = 0.020), HDL-C (*p* = 0.028), LDL-C (*p* = 0.034), and ApoA-1 (*p* = 0.027). BMI was relevant to TG (*p* = 0.042) and Glu (*p* = 0.015). Otherwise, in non-fasting status (2 h after breakfast), pregnancy complication was relevant to TC (*p* = 0.041), HDL-C (*p* = 0.014), Glu (*p* = 0.004). Delivery mode was relevant to TC (*p* = 0.012), HDL-C (*p* = 0.013), LDL-C (*p* = 0.026), ApoA-1 (*p* = 0.012), and sdLDL (*p* = 0.044). BMI was relevant to TG (*p* = 0.027).

**Table 3:** The correlation between serum lipid and characteristics of pregnant women and neonates

Characteristics	*P* value between serum lipids level and characteristics of pregnant women																	
	fasting									2-h after meal								
	TC	TG	HDL-C	LDL-C	ApoB	Glu	Lp(a)	ApoA1	sdLDL	TC	TG	HDL-C	LDL-C	ApoB	Glu	Lp(a)	ApoA1	sdLDL
age	0.708	0.275	0.534	0.561	0.948	0.274	0.736	0.955	0.232	0.779	0.155	0.845	0.733	0.757	0.221	0.847	0.672	0.082
BMI	0.326	0.042	0.263	0.265	0.335	0.015	0.583	0.886	0.318	0.486	0.027	0.377	0.340	0.484	0.311	0.592	0.592	0.095
Delivery mode	0.020	0.578	0.028	0.034	0.125	0.792	0.704	0.027	0.185	0.012	0.716	0.013	0.026	0.073	0.654	0.612	0.012	0.044
Pregnancy complications	0.165	0.042	0.073	0.131	0.248	0.014	0.222	0.502	0.212	0.041	0.062	0.014	0.061	0.079	0.004	0.161	0.137	0.339
Labour time	0.697	0.377	0.796	0.526	0.709	0.204	0.227	0.844	0.091	0.693	0.492	0.753	0.517	0.853	0.344	0.230	0.974	0.133
Perinatal outcomes	0.876	0.498	0.761	0.827	0.625	0.797	0.688	0.258	0.440	0.830	0.547	0.995	0.652	0.547	0.773	0.615	0.426	0.702
Apgar scores	0.562	0.876	0.236	0.816	0.848	0.128	0.382	0.107	0.180	0.989	0.886	0.545	0.959	0.866	0.443	0.401	0.431	0.236

## Discussion

Maternal fat accumulation and hyperlipidemia are characteristic features of maternal lipid metabolism [32]. The changes are essential for nutrient supply to fetal growth and development [33]. In our study, we found the higher level of lipid in pregnant women than controls, which was reported in previous studies [18, 34, 35].

Most studies have reported the significance of pregnant lipid level, but they ignored the influence of non-fasting [21, 36, 37]. In our study, we compared the differences between fasting and non-fasting lipid level of pregnant women. We found no differences of all lipids levels between fasting and non-fasting status in pregnant women. It showed that the non-fasting lipids levels can used to evaluate the serum lipid status of women instead of fasting lipids. Unlike the changes of lipids levels of controls after the meal (especially TG) in this study and other studies previous [38], the influence of intake may be ignored by the pregnant status and the results reported by other studies might not be diluted by the non-fasting state [21, 37].

We also found the elevated levels of most serum lipids including TC, LDL-C, ApoB, ApoA1 and sdLDL in pregnant women, which were also reported in previous studies [6, 10, 11, 39–41], but no significant difference was found for HDL-C concentration inconsistent with other researches [6, 9, 39]. It might be accounted for the trimester of pregnancy. Otherwise, we deemed the difference of TG concentration was no significant between pregnant women and controls. The appearance of differences of TG in fasting state might be affected by the huger state and energy over-consumption [42].

The clinical significance of serum lipids in pregnant women was also investigated in our study. The correlation between maternal conditions and lipids was statistically significant. Previous research has indicated that hyperlipidemia in pregnancy contributes to an increased morbidity of gestational diabetes mellitus (GDM) and preeclampsia [40]. Results of a few studies have suggested a concentration-dependent positive association between maternal TG, LDL-C and the risk of GDM and preeclampsia [14, 43–45]. GDM was also discovered to be associated with maternal HDL-C in several studies [16, 46–48]. Moreover, elevated maternal TC and TG levels are associated with an increased risk of preeclampsia and other pregnancy-related complications [21]. Chen Y et al. have suggested GDM patients have a tendency toward the predominance of small dense LDL particles, which may contribute to an increased risk for atherosclerosis and cardiovascular diseases (CVD) [49]. In our study, we have also confirmed fasting TG, non-fasting TC and HDL-C were related to pregnancy-related complications. But we did not found the correlation between LDL-C, sdLDL and pregnancy-related complications. These lipid and lipoprotein levels might indicate the tendency to gestational hyperlipidaemia and the related complications.The results also suggested no correlation between lipids levels and fetal adverse outcomes which was in contradiction to the previous researches [19, 37, 40]. However, our study had indicated indicators of serum lipids have affect the delivery mode. An increased level of lipids might cause an adverse delivery mode such as cesarean.

Some limitations existed in our study. One was that the case of samples was a little small, which might be a reason for the negative correlation between lipids and clinical significance. Future study should enlarge the samples. Another was the trimester included in our study is only the second trimester. The levels of serum lipids in other trimesters were unknown and should be investigated in future.

## Conclusion

In this study, we have suggested the non-fasting lipids detection might be used to estimate lipid metabolism in the second trimester pregnant women. However, the correlation between non-fasting lipids and lipid metabolism should be evaluated based on all-sided samples in the future. The non-fasting lipid and lipoprotein levels might indicate the tendency to gestational hyperlipidaemia.

## Abbreviations

ACC: American College of Cardiology
AHA: American Heart Association Guideline
ApoA-1: apolipoprotein A-1
ApoB: apolipoprotein B
BMI: Body Mass Index
CVD: cardiacvascular diseases
EAS/EFLM: European Atherosclerosis Society/European Federation of Clinical Chemistry and Laboratory Medicine
GDM: gestational diabetes mellitus
Glu: glucose
HDL: high density lipoproteins
LDL: low density lipoproteins
Lp(a): Lipoprotein (a)
NICE: National Institute of Clinical Excellence
SD: standard deviation
sdLDL: small dense LDL
TC: total cholesterol
TG: triglycerides
